# Can the Mean Nocturnal Baseline Impedance/Acid Exposure Time Ratio Serve as a Novel Parameter for the Definitive Diagnosis of Pathological Reflux?

**DOI:** 10.3390/jcm14186586

**Published:** 2025-09-18

**Authors:** Ayça Eroğlu Haktanır, Altay Çelebi

**Affiliations:** Department of Gastroenterology, Faculty of Medicine, Kocaeli University, Kocaeli 41001, Turkey; altaycelebi@yahoo.com

**Keywords:** acid exposure time, gastroesophageal reflux disease, Lyon Consensus 2.0, mean nocturnal baseline impedance, multichannel intraluminal impedance–pH, high-resolution esophageal manometry

## Abstract

**Background:** According to the Lyon Consensus 2.0, acid exposure time (AET) greater than 6% is considered definitive evidence of pathological reflux, while mean nocturnal baseline impedance (MNBI) serves as supportive evidence. Given the limitations in diagnostic accuracy when MNBI and AET are used separately, this study aimed to evaluate the MNBI/AET ratio as a potential novel parameter and determine its optimal cut-off value for improving diagnostic performance. **Methods:** We assessed patients with typical gastroesophageal reflux symptoms who completed standardized reflux questionnaires and underwent upper gastrointestinal endoscopy, high-resolution esophageal manometry, and 24 h multichannel intraluminal impedance–pH monitoring. Diagnoses were established based on the Lyon Consensus 2.0 and Chicago Classification v4.0 frameworks. **Results:** A total of 213 patients were included. Based on the Lyon Consensus 2.0, 66 patients (31%) were diagnosed with definite gastroesophageal reflux disease (GERD), 58 (27%) showed no evidence of reflux, and 89 (42%) had borderline or supportive findings. The cut-off value for MNBI to differentiate between patients with definitive reflux and those without reflux was ≤3040 Ω (AUC [95% CI]: 0.902 [0.836–0.948]; *p* < 0.001; sensitivity = 87.88%; specificity = 84.48%). Receiver operating characteristic (ROC) analysis revealed that an MNBI/AET ratio of ≤624 (95% CI: ≤607.5–≤624.28) most effectively distinguished patients with definitive GERD from those without reflux (AUC = 0.970; 95% CI: 0.937–0.988), demonstrating high sensitivity (98.5%) and specificity (98.3%). **Conclusions:** An MNBI/AET ratio ≤ 624 effectively differentiates patients with definitive GERD from those without reflux and may serve as a novel diagnostic parameter. Incorporating this ratio into clinical practice could enhance diagnostic accuracy for pathological reflux.

## 1. Introduction

Gastroesophageal reflux disease (GERD) is one of the most prevalent gastrointestinal (GI) disorders worldwide, with an estimated global prevalence of approximately 14%, rising to 21–25% in Europe and North America [1,2,3,4]. A diagnosis of GERD can be established by demonstrating typical mucosal injury on upper endoscopy, documenting pathological acid exposure through ambulatory pH monitoring, or observing symptom improvement following empirical treatment with proton pump inhibitors (PPIs) [5].

In patients with erosive esophagitis, ambulatory reflux monitoring demonstrates high diagnostic capabilities, with sensitivity ranging from 77% to 100% and specificity from 85% to 100%. However, the diagnostic accuracy declines significantly in patients with non-erosive reflux disease (NERD). Due to the limitations of conventional diagnostic modalities when used alone, there remains a need for more reliable and accurate diagnostic markers [6].

Initial evidence presented by Frazzoni et al. highlighted the diagnostic value of nocturnal baseline impedance (MNBI) in enhancing the sensitivity of multichannel intraluminal impedance–pH (MII-pH) monitoring for the detection of GERD [7,8,9,10]. These findings contributed to the development of the first Lyon Consensus in 2018, which established objective criteria for diagnosing GERD [11]. Building on subsequent research and clinical experience, the revised Lyon Consensus 2.0, published in 2024, further refines this diagnostic framework by underscoring the relevance of MNBI and associated impedance-based parameters as complementary tools in terms of improving diagnostic precision in clinical practice [12]. MNBI, introduced as a supportive parameter in this consensus, is a widely accepted method for evaluating distal esophageal mucosal impedance. It reflects mucosal integrity, electrical conductivity, and esophageal clearance [11,13]. According to the Lyon Consensus 2.0 [12], the presence of Los Angeles (LA) grade B, C, or D esophagitis [14]; a biopsy-proven Barrett’s esophagus; peptic strictures on upper GI endoscopy; or an AET > 6% during 24 h pH monitoring constitutes conclusive evidence of GERD. Conversely, an AET < 4%, a reflux episode (RE) count < 40, and an MNBI > 2500 Ω are considered evidence against GERD. Findings such as LA grade A esophagitis, a hiatal hernia, an AET between 4 and 6%, between 40 and 80 REs, an MNBI < 2500 Ω, or ineffective esophageal motility (IEM) determined via high-resolution esophageal manometry (HREM) are regarded as borderline or supportive evidence [12].

Importantly, the AET can vary significantly from day to day, even in patients with endoscopic evidence of esophagitis, which may lead to inconsistent diagnostic outcomes [15,16]. This variability has prompted interest in mucosal impedance metrics such as MNBI, as these parameters may offer a more stable and reliable assessment, particularly in cases where traditional diagnostic methods yield inconclusive results [9,17]. Recent studies have investigated the utility of MNBI as a surrogate marker of esophageal mucosal integrity and have explored its incorporation into diagnostic algorithms alongside the AET [8,16,17,18,19,20,21]. However, there is currently no consensus on the efficacy of combining these parameters into a unified metric to enhance diagnostic precision. Addressing this gap, the present study aimed to identify the optimal MNBI cut-off value for differentiating patients with definitive GERD from those with no evidence of reflux, as defined by the Lyon Consensus 2.0 [12].

To our knowledge, this is the first study to propose the MNBI/AET ratio as a composite diagnostic marker for GERD. We hypothesize that this ratio, by integrating both acid exposure (reflected by the AET) and mucosal integrity (represented by MNBI), can capture both functional and structural aspects of reflux severity, thereby enhancing diagnostic accuracy. Recognizing the limitations of the AET and MNBI when used independently, in this study, we aim to evaluate the MNBI/AET ratio as a novel parameter and to establish its optimal cut-off for reliably distinguishing patients with definitive GERD from those with no evidence of reflux, in accordance with the Lyon Consensus 2.0.

## 2. Materials and Methods

This study was conducted in accordance with the Declaration of Helsinki, Good Clinical Practice guidelines, and all applicable regulatory standards, including the Turkish Regulation on Clinical Trials (Official Gazette No: 28617, 2013) and the guidelines of the Turkish Medicines and Medical Devices Agency (TİTCK). Patient data were handled according to the Personal Data Protection Law (KVKK) to ensure confidentiality and privacy. Ethical approval was obtained from the institutional review board, i.e., the Clinical Research Ethics Committee of Kocaeli University Faculty of Medicine (protocol no. 2024/412; approval date: 10 October 2024), and written informed consent was collected from all participants prior to the study procedures.

Between January 2019 and January 2021, a total of 227 consecutive adult patients (aged > 18 years) presenting with typical reflux symptoms at the Gastroenterology outpatient clinic at Kocaeli University were evaluated. All patients completed a validated GERD questionnaire [22] and underwent upper GI endoscopy performed by a single physician (Celebi A). Those referred to the motility laboratory for physiologic reflux assessment were considered for inclusion. Patients were excluded if they had a history of upper GI surgery, esophagogastric junction outflow obstruction (EGJOO), major esophageal motility disorders such as achalasia (as identified by high-resolution manometry) [23], or a 24 h impedance–pH monitoring [24] duration of less than 22 h. Additionally, to avoid confounding factors that may affect MNBI values, patients with other esophageal pathologies known to present with a low MNBI, such as eosinophilic esophagitis (EoE) [25], were also excluded based on endoscopic and manometric findings. After applying the exclusion criteria, 213 eligible patients were included in the final analysis.

### 2.1. Clinical Assessment and Procedures

Demographic and clinical data (comorbidities, medication use, history of smoking/alcohol, and previous surgeries), as well as symptom characteristics (frequency, severity, and duration), were collected using the validated GERD questionnaire [22]. Endoscopic esophagitis severity was graded according to the LA classification system [14]. EoE was excluded based on endoscopic findings and, when clinically indicated (e.g., by the presence of dysphagia, esophageal rings, or linear furrows), confirmed via histological examination of esophageal biopsies [25].

### 2.2. High-Resolution Esophageal Manometry (HREM)

HREM was performed using a 32-channel solid-state catheter (Medical Measurement Systems B.V. [MMS], Enschede, The Netherlands) and interpreted in accordance with the Chicago Classification version 4.0 [23]. Patients diagnosed with EGJOO or major motility disorders such as achalasia were excluded based on the HREM results.

### 2.3. Multichannel Intraluminal Impedance–pH (MII-pH) Monitoring

Application of PPIs and H2 receptor antagonists was discontinued at least 10 days prior to MII-pH monitoring. Digitrapper MII-pH catheters (Medtronic, Minneapolis, MN, USA) were used, featuring a 6F (2 mm diameter) probe with five distal and three proximal impedance electrodes (spaced 2 cm apart) and a pH sensor located 5 cm above the lower esophageal sphincter (LES). After calibrating the pH sensor in accordance with manufacturer guidelines, transnasal insertion was performed with the pH sensor positioned 5 cm above the LES, as determined via prior HREM [23].

Impedance electrodes were placed to detect changes at 3, 5, 7, 9, 15, and 17 cm above the LES. Patients were instructed to use event markers to log meal times, posture changes, and symptom occurrences. Recordings were maintained for at least 22 h and up to 24 h. To ensure consistency, all MNBI measurements were performed manually by a single experienced physician (Haktanir A.E). Automated analysis was performed using the Digitrapper reflux reader software v6.1 (Medtronic), followed by manual confirmation. The MNBI was calculated by averaging impedance values from the distal esophageal channel (typically channel Z5, located 5 cm above the LES during three separate 10 min intervals at around 1:00 a.m., 2:00 a.m., and 3:00 a.m., specifically during periods without swallowing or RE [11,12,26]. We selected channel Z5 for measurement because AET is also assessed at this level, allowing for consistent and comparable analysis between the two parameters. All impedance–pH monitoring was conducted using the same catheter type and analysis system. The catheter included impedance sensors spaced at fixed intervals, and the MNBI measurements were consistently taken from the distal channel as determined by prior HREM. This standardization in catheter type and sensor positioning aimed to reduce technical variability and improve measurement reliability.

### 2.4. Classification Based on Lyon Consensus 2.0

Patients were classified into three groups using data from upper GI endoscopy, MII-pH, and HREM, in accordance with the Lyon Consensus 2.0 [12]. Conclusive evidence for GERD included LA grade B–D esophagitis, biopsy-proven Barrett’s esophagus, peptic stricture, or AET > 6%. Borderline evidence included LA grade A esophagitis, an AET between 4% and 6%, or between 40 and 80 REs. Supportive evidence in patients not meeting criteria for conclusive GERD included MNBI < 1500 Ω, the presence of an HH, >80 REs, a positive symptom association probability (SAP) or symptom index (SI), or ineffective esophageal motility (IEM) on HREM. SAP was considered positive if ≥95%, and SI was deemed positive if ≥50%, in accordance with established guidelines [12,26]. Evidence against GERD included normal endoscopy without esophagitis, an AET < 4%, < 40 REs, MNBI > 2500 Ω, and negative symptom association [12].

**Figure 1:**
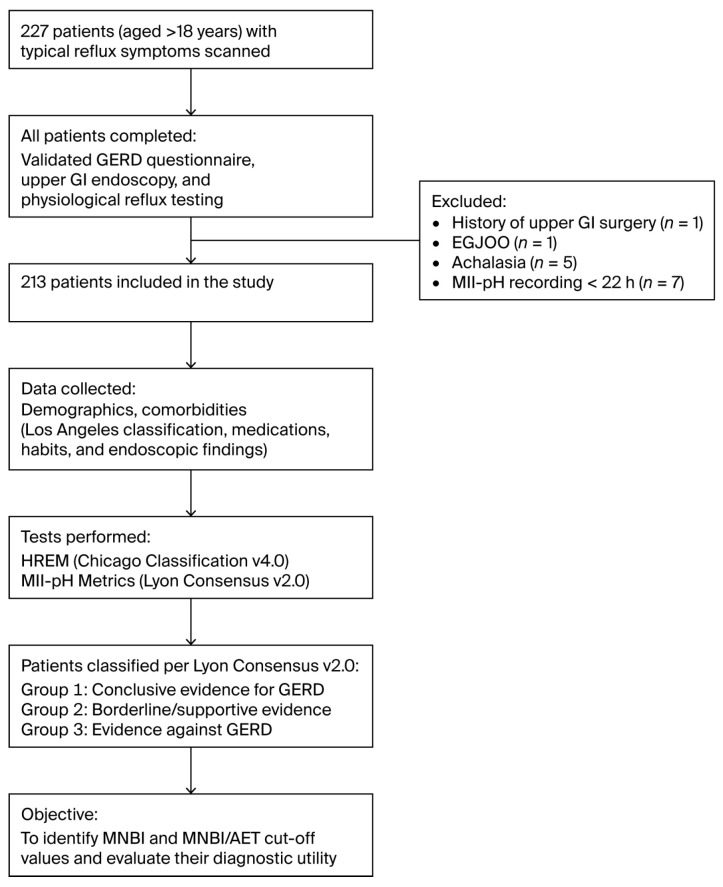
Study design diagram. Abbreviations: GERD, gastroesophageal reflux disease; GI, gastrointestinal; HREM, high-resolution esophageal manometry; EGJOO, esophagogastric junction outflow obstruction; MII-pH, multichannel intraluminal impedance–pH; MNBI, mean nocturnal baseline impedance; AET, acid exposure time.

We compared patients with conclusive evidence of pathological reflux to those with evidence against reflux to determine a cut-off value for the MNBI that could differentiate between the two groups. Given the limited sensitivity and specificity of the MNBI and AET when used separately, we identified the optimal cut-off for the MNBI/AET ratio as a new diagnostic marker aimed at improving diagnostic precision. A study flow chart is presented in Figure 1.

### 2.5. Statistical Analysis

All statistical analyses were performed using IBM SPSS Statistics, version 29.0 (IBM Corp., Armonk, NY, USA). The normality of the data distribution was evaluated using both the Kolmogorov–Smirnov and Shapiro–Wilk tests. Since most continuous variables did not follow a normal distribution, they are presented as medians with interquartile ranges (IQRs), while categorical variables are summarized as frequencies and percentages.

Comparisons between groups were conducted using the Kruskal–Wallis test, followed by Dunn’s post hoc test for pairwise comparisons. Associations between categorical variables were analyzed using the Chi-square test with Bonferroni correction to adjust for multiple comparisons.

Receiver operating characteristic (ROC) curve analysis was employed to assess the diagnostic performance of selected parameters by calculating the area under the curve (AUC), sensitivity, specificity, and optimal cut-off values. The cut-off value for the MNBI/AET ratio was selected based on the maximum Youden index derived from the ROC curve. Statistical significance was defined as a two-tailed *p*-value of < 0.05. A post hoc power analysis using G*Power 3.1 confirmed that the sample size (*n* = 213) provided sufficient power (80%) to detect a statistically significant difference in the MNBI and MNBI/AET ratio between groups, assuming a medium effect size and a significance level of α = 0.05.

## 3. Results

### 3.1. Study Population

A total of 227 consecutive patients were initially enrolled. After excluding individuals with a history of upper-GI surgery (*n* = 1), EGJOO (*n* = 1) and/or achalasia (*n* = 5) and those with incomplete 24 h impedance–pH monitoring records (<22 h; *n* = 7), data from 213 patients who met the inclusion criteria were analyzed.

Among the participants, 104 (49%) were female and 109 (51%) were male. The median age was 43 years (36–54), and the median body mass index (BMI) was 25.7 kg/m^2^ (23–28).

### 3.2. Symptoms and Comorbidities

**Figure 2:**
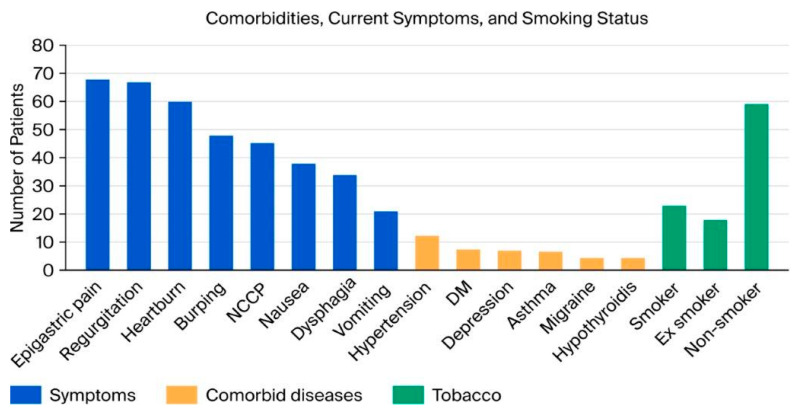
Distribution of comorbidities and presenting symptoms among study participants. Abbreviations: NCCP, non-cardiac chest pain; DM, diabetes mellitus.

The presenting symptoms, in descending order of frequency, included epigastric pain in 145 (68%) patients, regurgitation in 143 (67%), heartburn in 128 (60%), frequent belching in 103 (48%), non-cardiac chest pain in 95 (45%), nausea in 80 (38%), dysphagia in 73 (34%), and vomiting in 45 (21%). Comorbid conditions were present in 88 (41%) patients. These included diabetes mellitus in 16 (7.5%), asthma in 14 (6.6%), hypothyroidism in 9 (4.2%), allergy in 8 (3.8%), hyperlipidemia in 5 (2.3%), depression in 15 (7%), migraines in 10 (4.3%), coronary artery disease in 5 (2.3%), hypertension in 26 (12.2%), and rheumatoid arthritis in 5 (2.3%). Regarding lifestyle factors, 125 patients (59%) were non-smokers, 49 (23%) were current smokers, and 39 (18%) were former smokers (Figure 2). Alcohol consumption was reported by 28 patients (13%).

### 3.3. Endoscopic and Manometric Findings

Endoscopic evaluation based on the LA classification revealed normal esophageal mucosa in 181 patients (85%), with esophagitis observed as LA grade A in 20 patients (9.4%), LA grade B in 8 patients (3.3%), and LA grade C in 4 patients (2.3%). Other findings included hiatal laxity in 68 patients (32%) and a sliding HH (<3 cm) in 21 patients (10%).

The median duration of MII-pH monitoring was 23:15 h (22.28–23.53). The AET was <4% in 122 (57%) patients, 4–6% in 29 (14%), and >6% in 62 (29%). MNBI was <1500 Ω in 23 (11%), between 1500 and 2500 in 60 (28%), and >2500 in 130 (61%) patients. The number of REs was <40 in 156 (73%), between 40 and 80 in 45 (21%), and >80 in 12 (6%) patients. HREM findings included a median supine integrated relaxation pressure (IRP) of 7 mmHg (4–12), supine distal contractile integral (DCI) of 668 mmHg·s·cm (369–1046), contractile front velocity of 3.2 cm/s (0.5–4), peristaltic break of 1.8 cm (0.6–4), supine distal latency of 6.9 s (6.4–7.79), and LES resting pressure of 19 mmHg (11–25).

Based on the Lyon Consensus 2.0 criteria, 66 patients (31%) were classified as having conclusive evidence of pathological reflux, 58 patients (27%) as having evidence against pathological reflux, and 89 (42%) as having borderline and/or supportive evidence. Among these 89 patients, 31 exhibited both borderline and supportive findings, 9 had isolated borderline findings, and 49 had isolated supportive findings (Table 1). There were no statistically significant differences in gender or body mass index among the three groups. In patients without endoscopic esophagitis, 58 (32%) were classified as having no evidence of reflux, 77 (42.5%) fell into the borderline/supportive group, and 46 (25.4%) had conclusive reflux. Among those with LA grade A esophagitis, 12 (60%) belonged to the borderline/supportive group, while 8 (40%) were in the conclusive reflux group.

Across all patients, the median AET was 2.9% (0.7–6.3), there were 26 REs (12–43), and the MNBI was 3040 Ω (2125–4255). In the non-reflux group, the median AET was 0.55% (0–3.9), the number of REs was 12 (0–51), and MNBI was 4180 Ω (1580–8420). Conversely, patients in the conclusive reflux group had a median AET of 8.6% (0.1–39), 36 REs (2–138), and MNBI of 1880 Ω (670–4760). Statistical analysis demonstrated significantly higher AET and RE values and markedly lower MNBI measurements in patients with confirmed reflux compared to the non-reflux cohort (*p* < 0.001 for all comparisons) (Table 2).

Among the 213 patients initially evaluated, 4 were unable to complete HREM because of intolerance, leaving 209 patients for final analysis. Within this cohort, 68% demonstrated normal manometric findings, while 32% were diagnosed with IEM. However, no statistically significant differences were observed in AET and MNBI between patients with normal and impaired esophageal motility (*p* = 0.183 and *p* = 0.86, respectively). In the conclusive reflux group, the median values for supine DCI, peristaltic break length, IRP, and LES resting pressure were 531 mmHg·s·cm (250–944), 3.1 cm (0.83–5.73), 7 mmHg (3–12), and 18 mmHg (9.25–23), respectively. In contrast, the non-reflux group exhibited median values of 733 mmHg·s·cm (466–1298), 1.3 cm (0.2–2.9), 7 mmHg (5–12), and 20 mmHg (14–25), respectively. Compared to the non-reflux group, patients with conclusive reflux had significantly lower DCIs and LES resting pressures (*p* < 0.001 and *p* = 0.008, respectively), along with significantly longer peristaltic breaks (*p* < 0.001). When patients were stratified according to MNBI values, in the MNBI < 1500 Ω group (*n* = 23), 78% (*n* = 18) were in the conclusive reflux group and 22% (*n* = 5) in the borderline/supportive group (*p* = 0.001); in the MNBI 1500–2500 Ω group (*n* = 60), 10% (*n* = 6) were in the non-reflux group, 33.3% (*n* = 20) in the borderline/supportive group, and 56.7% (*n* = 34) in the conclusive reflux group; and in the MNBI > 2500 Ω group (*n* = 130), 40% (*n* = 52) were in the non-reflux group, 49.2% (*n* = 64) in the borderline/supportive group, and 10.8% (*n* = 14) in the conclusive reflux group.

**Figure 3:**
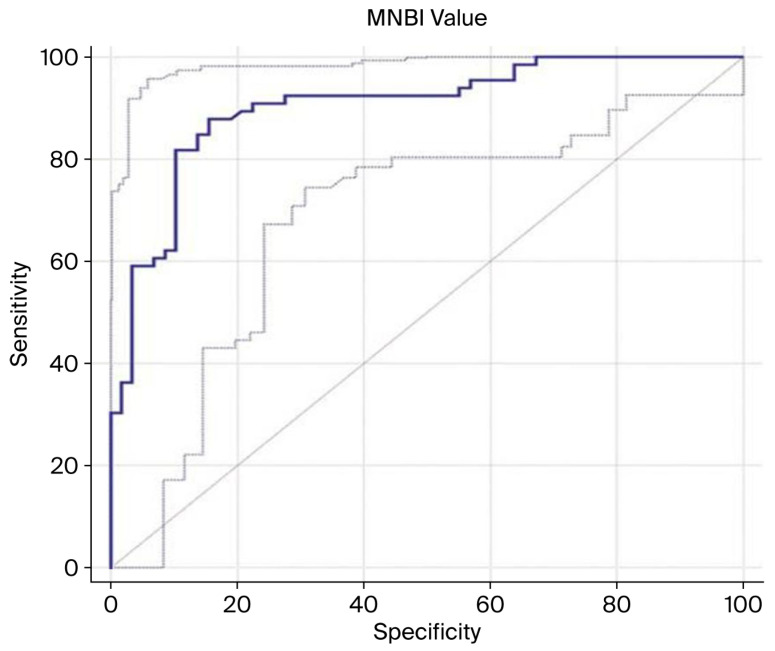
Comparison between patients with definitive reflux (*n* = 66) and those without reflux (*n* = 58). The dark-blue line represents the ROC curve of the MNBI, and the gray line shows the 95% confidence interval. The diagonal line a diagnostic test with the lowest discriminatory ability, which is no better than chance, area under the curve (AUC) = 0.5. The optimal MNBI cut-off value was ≤3040 Ω, with an area under the receiver operating characteristic (ROC) curve (AUC) of 0.902 (95% confidence interval [CI]: 0.836–0.948), sensitivity of 87.88%, and specificity of 84.48% (*p* < 0.0001). Abbreviations: AUC, area under the curve; CI, confidence interval.

Receiver operating characteristic (ROC) curve analysis was employed to evaluate the diagnostic performance of MNBI in distinguishing patients with conclusive gastroesophageal reflux from those without reflux. The analysis identified an optimal MNBI threshold of ≤3040 Ω, which demonstrated good discriminative ability, as reflected by an area under the curve (AUC) of 0.843 (95% confidence interval: 0.787–0.889; *p* < 0.0001). At this cut-off value, the sensitivity was 59.1%, indicating the proportion of true positive cases correctly identified, while the specificity was 89.8%, reflecting the ability to correctly exclude non-reflux cases (Figure 3).

**Figure 4:**
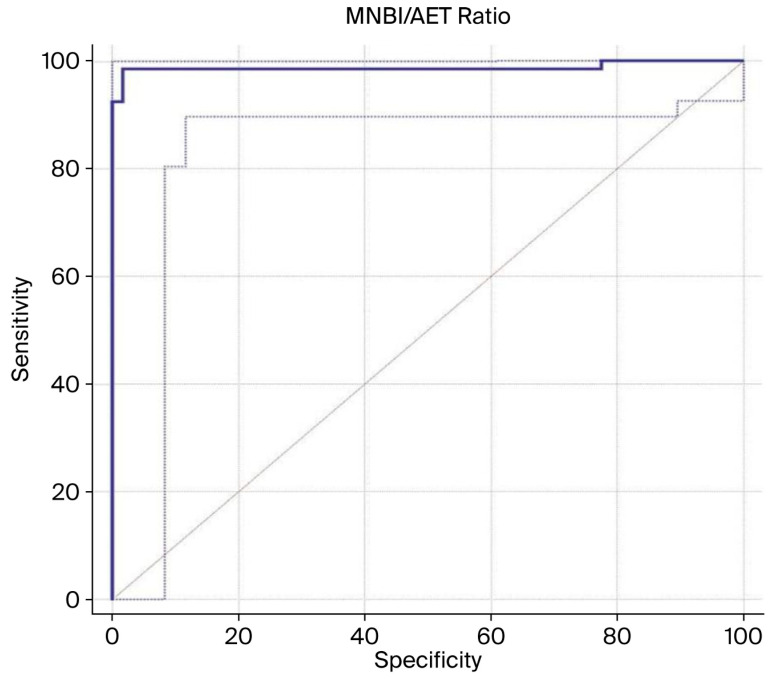
Receiver operating characteristic (ROC) curve analysis comparing patients with definitive reflux (*n =* 66) with those without reflux (*n* = 58). The dark-blue line represents the actual ROC curve of the MNBI/AET ratio, while the gray line illustrates the 95% confidence interval. The diagonal line a diagnostic test with the lowest discriminatory ability, which is no better than chance, area under the curve (AUC) = 0.5. The area under the ROC curve (AUC) was 0.987 (standard error: 0.0118; 95% CI: 0.948–0.999; *p* < 0.0001), suggesting excellent diagnostic performance. The optimal cutoff value for the MNBI/AET ratio, based on the Youden index (J = 0.9676), was ≤624.28 (95% CI: ≤607.5 ≤624.28), yielding a sensitivity of 98.48% and a specificity of 98.28%. Abbreviations: AUC, area under the curve; CI, confidence interval; ROC, receiver operating characteristic.

Considering the modest sensitivity and specificity of both AET and MNBI when used individually for diagnostic purposes, we hypothesized that their combined use might enhance diagnostic accuracy for reflux disease. Accordingly, the MNBI/AET ratio was computed for each patient, and its effectiveness in diagnosing conclusive pathological reflux was statistically evaluated. ROC curve analysis identified an optimal MNBI/AET ratio cut-off of ≤624.28 (95% CI: ≤607.5–≤624.28) for distinguishing patients with conclusive pathological reflux from those without reflux, yielding excellent diagnostic performance with an AUC of 0.987 (95% CI: 0.948–0.999; *p* < 0.001), a sensitivity of 98.48%, a specificity of 98.28%, a positive predictive value (PPV) of 98.5%, and a negative predictive value (NPV) of 98.3% (Figure 4).

These results indicate that the MNBI/AET ratio provides significantly improved diagnostic accuracy compared to the MNBI or AET alone and can reliably differentiate patients with definitive reflux from non-reflux cases. Upon applying this cut-off across the study cohort, 21 patients (23.6%) from the borderline and/or supportive group met the criteria for reclassification into the conclusive reflux group. Of these, 20 patients had borderline AET values (4–6%), and one had an AET of 3.7%. All 21 patients exhibited at least one supportive or borderline parameter. Among them, 15 patients had MNBI values below 2500 Ω and 19 had MNBI values below 3040 Ω. Importantly, none of the patients classified in the non-reflux group exhibited an MNBI/AET ratio ≤ 624. Detailed clinical and diagnostic characteristics of these 21 patients are presented in Table 3.

## 4. Discussion

In this study, we evaluated upper GI endoscopy, HREM, and 24 h multichannel intraluminal impedance–pH (MII-pH) monitoring data from 213 patients presenting with typical reflux symptoms, in accordance with the Lyon Consensus 2.0 criteria. Based on these assessments, 66 patients (31%) were diagnosed with conclusive pathological reflux, 58 patients (27%) had findings that argued against reflux disease, and 89 patients (42%) exhibited either borderline or supportive evidence of reflux. Through ROC analysis, we identified an MNBI threshold of <3040 Ω as the optimal cut-off for distinguishing patients with conclusive reflux from those without reflux. Furthermore, we demonstrated that the MNBI/AET ratio, with an optimal cut-off value of ≤624, provided excellent diagnostic performance—achieving a sensitivity of 98.48% and specificity of 98.28%.

Since impedance–pH monitoring depends on detecting intraluminal acid and non-acid REs over a relatively short period of 24 to 48 h, its diagnostic sensitivity and specificity are considerably limited by day-to-day variability in reflux activity [15,16]. As an alternative, wireless pH monitoring offers a more tolerable option for patients who are unable to undergo transnasal catheter-based studies and allows for an extended recording duration of 3 to 4 days [15]. However, studies have shown that, with wireless pH monitoring over 72 to 96 h, an abnormal AET is observed on only one of the two monitored days in approximately 24% to 32% of patients, further compromising diagnostic reliability [16]. Although the AET remains the primary parameter for detecting pathological reflux, it is important to note that up to one-third of patients with reflux esophagitis may have AET values within the normal range [7,11,15,16,26]. In cases where AET results are inconclusive, the total number of daily REs may be considered; however, reliance on automated analysis software carries the risk of overestimating reflux events [27].

The SAP and SI are commonly used impedance metrics to evaluate the temporal relationship between REs and symptom occurrence. However, the sensitivity of both may be limited in some patients who report few symptoms during the 24 h monitoring period, reducing the reliability of these parameters [28].

Mucosal impedance, especially MNBI, has gained recognition as a sensitive biomarker reflecting esophageal mucosal integrity and damage caused by acid exposure [19,26]. MNBI complements traditional reflux metrics by offering improved diagnostic clarity, particularly in cases with normal or borderline AETs, a role supported by the Lyon Consensus 2.0, which classifies MNBI as either supportive or reflux-excluding evidence [12,20].

Manual MNBI measurement provides enhanced diagnostic precision by enabling expert-guided exclusion of artifact-laden segments, thereby improving data quality—an approach particularly valuable in settings lacking reliable automation [29]. In this study, all impedance–pH tracings were manually reviewed in 2-min time windows by a single experienced physician, ensuring consistent and reproducible evaluation across all recordings. The MNBI values were calculated during nocturnal recumbency, excluding periods affected by swallowing, reflux events, or artifacts. While manual assessment may limit inter-observer generalizability, it allowed for accurate identification of physiologically stable periods. Future research should focus on developing standardized, automated, or consensus-based protocols to help expand clinical applicability and increase reproducibility.

Multiple studies have demonstrated significantly lower MNBI values in patients with confirmed pathological reflux compared to those with functional heartburn (FH), indicating its utility in differentiating GERD phenotypes [19,30,31,32,33]. For instance, MNBI values typically fall below 1500 Ω in GERD patients, whereas asymptomatic individuals tend to have values exceeding 2500 Ω [8,9,10,19,20,26]. Moreover, MNBI has been proposed as a predictor of treatment response, especially in PPI-refractory cases, further emphasizing its clinical value [34]. A large multicenter investigation by Frazzoni et al. revealed that MNBI and the post-reflux swallow-induced peristaltic wave (PSPW) index vary progressively across GERD phenotypes, with the lowest MNBI recorded in NERD patients (1378 Ω), intermediate values in reflux hypersensitivity patients (2274 Ω), and the highest in FH patients (3445 Ω) [8]. These findings were supported by a retrospective study from China, which demonstrated a stepwise increase in both MNBI and the PSPW index across reflux esophagitis, NERD, RH, and FH, underscoring the diagnostic relevance of these parameters in phenotype differentiation [30]. Additionally, MNBI and the PSPW index have shown diagnostic value in patients presenting with extra-esophageal symptoms, broadening their applicability [34]. Patients with confirmed pathological reflux consistently exhibit lower MNBI values than those with RH or FH, with RH patients showing lower MNBI than FH patients [30,32,33,35]. Despite these clear distinctions, the Lyon Consensus 2.0 does not yet provide phenotype-specific MNBI thresholds, highlighting the need for further refinement in these diagnostic criteria [12].

Our study identified a distal MNBI cut-off of 3040 Ω that effectively discriminates patients with pathological reflux from those without, achieving robust diagnostic accuracy (AUC: 0.902, 95% CI: 0.836–0.948; *p* < 0.0001) with an 87.88% sensitivity and 84.48% specificity. This threshold is higher than the 2500 Ω cut-off suggested by the Lyon Consensus to indicate preserved mucosal integrity, suggesting regional or methodological differences may influence the optimal MNBI values. Supporting this, multiple studies from European and Asian cohorts report varying MNBI cut-offs: Frazzoni et al. recommended values of 2292 Ω for MNBI and 61% for the PSPW index [8], while other multicenter analyses proposed thresholds of around 2000 Ω and 50%, respectively [10]. A Chinese study identified MNBI and PSPW cut-offs of approximately 1942 Ω and 27.5%, respectively, to differentiate FH from other reflux subtypes [30]. Other investigations reported MNBI thresholds below 1865 Ω [20] and 2292 Ω for pathological reflux diagnosis [21], with some suggesting values below 1000 Ω to distinguish NERD from FH, which often shows MNBI values above 3000 Ω [33,36]. A recent prospective study conducted by Liu et al. explored the predictive value of the MNBI and PSPW indices in determining PPI responses in patients with reflux hypersensitivity [37]. Their findings demonstrated that both lower MNBI values (1866.68 ± 390.62 Ω) and PSPW (47.05 ± 4.43) index values were significantly associated with PPI treatment response [37]. Notably, a Korean cohort exhibited an optimal MNBI threshold of 2167 Ω, significantly above the Lyon 2.0 criteria [38]. This collective evidence further supports the observation that there is notable geographic variability in MNBI values, with Asian and South African populations generally exhibiting higher distal MNBI values than Europeans, likely reflecting differences in reflux prevalence, mucosal injury severity, diet, genetics, and cultural habits owing to technical factors [8,10,12,13,21,32,33,36,39].

In addition to population-specific factors, significant variability in MNBI values has been linked to differences in types of catheters and their positioning during measurement. Moreover, the clinical application of the MNBI is complicated by methodological inconsistencies across studies and institutions, such as variations in impedance channel placement and the selection of nocturnal time intervals, all of which can substantially impact absolute MNBI values and the diagnostic thresholds derived from them. Several studies have shown that MNBI values obtained using MMS catheters, particularly at the 5 cm level above the LES, tend to be higher than those recorded with other systems [39]. In addition, there is currently no consensus in the literature regarding the optimal anatomical level for MNBI measurement. While some researchers have assessed the MNBI at 3 cm above the LES, others have used the 5 cm point, and values have been recorded at various locations, ranging from 3 to 17 cm above the LES. These inconsistencies in measurement technique may influence diagnostic outcomes. In this study, the MNBI was calculated by averaging impedance values obtained from three artifact-free, physiologically stable 10-min nocturnal periods recorded at 1:00 a.m., 2:00 a.m., and 3:00 a.m., measured at 5 cm above the LES, in accordance with established recommendations [12,29,40]. To improve the clinical utility and comparability of impedance-derived parameters such as the MNBI/AET ratio, measurement protocols must be standardized across centers and equipment platforms. Future international consensus initiatives should aim to establish uniform guidelines for the acquisition, analysis, and reporting of impedance–pH data.

Taken together, these findings underscore the critical influence of both population characteristics and methodological variability on MNBI thresholds. Such factors contribute substantially to the heterogeneity observed across studies and highlight the need for population-specific diagnostic criteria to improve clinical accuracy and relevance [39]. Accordingly, the higher cut-off identified in our study should be interpreted within the context of its specific protocol and patient population. It is essential to conduct prospective, multicenter studies employing standardized methodologies in order to validate and generalize these thresholds across broader clinical settings.

Although MNBI is regarded as a relatively stable metric less influenced by short-term physiological fluctuations, its values may be reduced not only by acid-induced mucosal injury but also by other esophageal conditions associated with epithelial compromise, such as EoE, achalasia, or infectious esophagitis,—thereby potentially limiting its diagnostic specificity when employed as a standalone metric [25,36]. Moreover, the Lyon Consensus 2.0 [12] recognizes that borderline MNBI values may support a diagnosis of GERD but, on their own, are insufficient for definitive confirmation. On the other hand, AET quantifies acid exposure but may underestimate disease severity in patients with increased mucosal sensitivity or intermittent reflux [27]. To address this diagnostic gap, considering that MNBI and AET values vary across populations because of factors such as diet and mucosal characteristics, and AET can fluctuate significantly from day to day [15,16,39,41], we hypothesized that relying on a single 24-h MII-pH measurement made with currently available devices may result in missing pathological acid reflux cases present on other days. By integrating mucosal integrity and acid burden into a single metric, the MNBI/AET ratio offers a more stable and clinically relevant diagnostic tool that minimizes the impact of daily fluctuations. This composite parameter enhances diagnostic consistency and accuracy, particularly for patients with borderline or inconclusive findings, thereby improving reflux evaluation across diverse patient populations. While the initial results from our single-center study are promising, future multicenter validation studies are needed to assess the performance and stability of this ratio in broader clinical settings.

The Lyon Consensus 2.0 has retired the PSPW index as a routine diagnostic criterion for definitive GERD diagnosis, recognizing it primarily as a research tool for phenotyping refractory GERD owing to its variable normative thresholds and complex calculation [12]. Consequently, the PSPW index was not incorporated in our study. In contrast, the MNBI offers more straightforward and longitudinal evidence of mucosal integrity impairment, making the MNBI/AET ratio a practical and robust parameter that may enhance diagnostic accuracy in clinical practice without the limitations associated with PSPW.

In our analysis, ROC curve evaluation identified an optimal MNBI/AET ratio cut-off of ≤624.28 for distinguishing patients with conclusive pathological reflux from those without objective evidence of reflux. This threshold demonstrated excellent diagnostic performance, with a sensitivity of 98.48% and specificity of 98.28%. When applied to the entire cohort, 23.6% (*n* = 21) of patients initially categorized within the borderline or supportive evidence group were reclassified as having conclusive pathological reflux. Of these reclassified patients, 20 had AET values within the borderline range (4–6%), and one had an AET of 3.7%. Notably, 16 of these patients had MNBI values below 2500 Ω and 19 had values below 3040 Ω. These results suggest that individuals with a borderline AET and reduced MNBI may indeed represent undiagnosed cases of pathological GERD. Our findings are in line with those reported by Rengarajan et al., who demonstrated that patients with either an abnormal (>6%) or borderline (4–6%) AET, when accompanied by a low MNBI, were more likely to respond favorably to anti-reflux therapy [27]. Similarly, a separate study showed that patients with MNBI values below 2292 Ω had significantly greater symptom improvement following PPI therapy than those with higher MNBI levels (60.1% vs. 17.4%) [29]. Although the MNBI/AET ratio demonstrated excellent diagnostic performance in our internal analysis, external validation in independent cohorts is warranted before clinical application owing to potential geographic, demographic, and methodological variability. Based on this ratio, 21 patients were reclassified from borderline to definitive GERD; Subsequently, a retrospective review of electronic medical records was conducted, yielding treatment response data for 20 patients, 18 of whom exhibited symptomatic improvement following PPI therapy. Future prospective studies with integrated outcome tracking will be essential to determine whether reclassification based on this ratio corresponds to a meaningful clinical benefit, thereby validating its practical utility.

The incorporation of the MNBI/AET ratio into routine clinical workflows may pose certain challenges. First, manual calculation of the MNBI—currently required in the absence of software automation—requires trained personnel and additional analysis time, with manual measurement taking approximately 30–40 s per case, which, despite being a relatively short analysis time, may limit feasibility in high-volume centers. Additionally, differences in catheter types, impedance measurement sites, and software algorithms across institutions could lead to variability in MNBI values and complicate uniform adoption of the ratio. The cost-effectiveness of implementing this parameter also remains uncertain, as formal health-economic analyses have yet to be conducted. To ensure clinical translation, future studies should assess not only diagnostic accuracy but also operational feasibility, resource allocation, and the potential for integration into automated analysis platforms.

Our study has several limitations. Primarily, its retrospective, single-center design limits causal inference and may reduce the generalizability of the results. Moreover, the cohort, though well-characterized, included patients referred for MII-pH testing because of persistent or complex symptoms, introducing selection bias and possibly underrepresenting certain GERD phenotypes—especially given the exclusion of incomplete studies and major motility disorders. Additionally, the lack of randomization and prospective follow-ups hinders the evaluation of long-term outcomes, such as symptom resolution and treatment response.

We explored potential confounding factors that might influence the performance of the MNBI/AET ratio, including age, BMI, sex, presence of comorbidities, and duration of comorbidities. Among these, only sex showed a statistically significant difference. However, when we attempted to calculate sex-specific cut-off values, the sample size within each subgroup became too small to provide reliable estimates. Therefore, we reported the overall cut-off. Although MNBI values were manually calculated by a single experienced physician following a standardized protocol, the lack of inter-observer comparison limits the reproducibility of these findings. To ensure MNBI measurements are reliable, future studies should incorporate blinded assessments conducted by multiple observers. Moreover, the diagnostic utility of the MNBI/AET ratio requires validation in larger, prospectively designed, and more diverse patient populations. Further research should also establish subgroup-specific thresholds to enhance clinical applicability.

This study is the first to introduce and validate the MNBI/AET ratio, a novel impedance–pH parameter not currently incorporated into the Lyon Consensus 2.0 framework. Derived via ROC curve analysis, the MNBI/AET ratio demonstrated excellent diagnostic performance, characterized by high sensitivity and specificity in differentiating conclusive GERD from non-reflux cases.

By integrating AET and the MNBI into a single composite metric, the MNBI/AET ratio addresses limitations associated with evaluating these parameters in isolation. This novel ratio improves diagnostic clarity in cases with borderline acid exposure or inconclusive reflux evidence, where conventional metrics may yield ambiguous interpretations. Moreover, it accounts for inter-day variability in acid exposure and mucosal integrity, offering a more physiologically stable and clinically reliable indicator.

The MNBI/AET ratio constitutes a promising composite impedance–pH metric that may enhance the precision of diagnosing GERD. Its integration into clinical algorithms has the potential to support more individualized and timely treatment approaches. To ensure its broader applicability and diagnostic reliability, future studies should validate this parameter in larger, more diverse populations, including asymptomatic individuals and patients with overlapping esophageal conditions. Incorporating treatment outcomes such as symptom resolution, PPI responsiveness, and post-surgical follow-ups will be critical for assessing its clinical utility in guiding evidence-based interventions. Additionally, standardization of MNBI acquisition protocols—including catheter specifications, sensor positioning, and measurement criteria—will be essential to improve reproducibility and facilitate adoption into routine clinical practice. These steps are necessary to establish the MNBI/AET ratio as a robust and generalizable tool in the evolving diagnostic landscape of GERD.

## 5. Conclusions

This study identified an optimal MNBI/AET ratio cut-off value of ≤624.28, which demonstrated high diagnostic accuracy for distinguishing patients with conclusive pathological reflux from those without reflux, with a sensitivity of 98.48% and a specificity of 98.28%. Given its superior performance compared to MNBI or AET alone, the MNBI/AET ratio shows promise as a novel and reliable parameter in the evaluation of gastroesophageal reflux.

Importantly, this ratio may be particularly valuable in clinical practice for patients with borderline or inconclusive findings under the Lyon Consensus 2.0, where traditional metrics often fail to provide diagnostic certainty. Incorporating the MNBI/AET ratio into routine MII-pH analysis has the potential to enhance diagnostic confidence and improve patient stratification, thereby supporting more accurate and timely management decisions in individuals with suspected pathological reflux.

However, as this is a proof-of-concept study, further multicenter studies and external validations are necessary before the MNBI/AET ratio can be widely adopted as a clinical diagnostic tool. Future research should also focus on standardizing measurement protocols and evaluating the utility of this parameter across diverse patient populations and geographic regions.

## Figures and Tables

**Table 1:** Analysis results of patients based on the Lyon Consensus 2.0.

Results of Patients Based on the Lyon Consensus 2.0	*n* (%)
Conclusive evidence for pathological reflux	66 (31%)
AET > 6	62 (29.1%)
LA-B esophagitis	7 (3.3%)
LA-C esophagitis	4 (2.3%)
Evidence against pathologic reflux	58 (27%)
AET < 4	122 (57%)
REs < 40	156 (73%)
MNBI > 2500	130 (61%)
Patients with borderline and/or supportive evidence	89 (42%)
Only borderline evidence	9 (4.2%)
Only supportive evidence	49 (23%)
Both borderline and supportive evidence	31 (14%)

Abbreviations: AET, acid exposure time; LA, Los Angeles classification; LA-B, Los Angeles grade B; LA-C, Los Angeles grade C; MNBI, mean nocturnal baseline impedance; REs, reflux episodes; *n*, number of patients; %, percentage of patients.

**Table 2:** Comparison of pH–impedance parameters and MNBI values among patients categorized as non-reflux, borderline/supportive, and definitive reflux groups according to the Lyon Consensus 2.0.

Total Patients (*n* = 213)	No Reflux (*n* = 58)	Borderline/Supportive (*n* = 89)	Definitive Reflux (*n* = 66)	*p*-Value	Inter-Group Significance
MNBI (Ω)	4180 ^a^ (2580–8420)	3330 ^b^ (1000–6500)	1880 ^c^ (670–4760)	<0.001	c-b = 0.001 c-a = 0.001 b-a = 0.01
RE number	12 ^a^ (0–51)	28 ^b^ (0–160)	36 ^c^ (2–138)	<0.001	a-b = 0.001 a-c = 0.001 b-c = 0.196
AET% value	0.55 ^a^ (0–3.9)	1.9 ^b^ (0–6)	8.6 ^c^ (0.1–39)	<0.001	a-b = 0.002 a-c < 0.001 b-c < 0.001

Data are presented as median (quartiles) and interquartile range (IQR, 25th–75th percentile). MNBI, mean nocturnal baseline impedance (Ω); REs, reflux episodes; AET, acid exposure time. Superscript letters (a, b, c) indicate results of post hoc pairwise comparisons between groups. Within each row, values with different superscript letters are significantly different (*p* < 0.05).

**Table 3:** Detailed multichannel intraluminal impedance–pH (MII-pH) findings of patients from the borderline and/or supportive evidence group who were reclassified into the definitive reflux group based on an MNBI/AET ratio cut-off value of ≤624.28.

No.	MNBI/AET < 624	MNBI (Ω)	AET	RE	SA	Evidence Level (Lyon Consensus 2.0)
1.	315	1730	5.5	44	−	Borderline
2.	551	2480	4.5	60	+	Borderline and supportive
3.	574	3330	5.8	13	+	Borderline and supportive
4.	425	2420	5.7	41	−	Borderline
5.	416	1750	4.2	36	+	Borderline and supportive
6.	254	1450	5.7	24	+	Borderline and supportive
7.	563	2760	4.9	52	−	Borderline and supportive (IEM)
8.	605	3630	5.9	19	+	Borderline and supportive
9.	406	1910	4.7	31	−	Borderline and supportive (IEM)
10.	222	1000	4.5	89	−	Borderline and supportive
11.	318	1880	5.9	48	−	Borderline
12.	488	2150	4.4	34	+	Borderline and supportive
13.	453	2310	5.1	31	−	Borderline and supportive (IEM)
14.	541	2760	5.1	160	+	Borderline and supportive
15.	369	1550	4.2	60	+	Borderline and supportive
16.	480	1970	4.1	40	−	Borderline
17.	440	2200	5	40	−	Borderline and supportive (IEM)
18.	389	2220	5.7	60	+	Borderline and supportive
19.	534	2510	4.7	24	+	Borderline and supportive
20.	500	2800	5.6	14	−	Borderline and supportive (HH)
21.	427	1580	3.7	38	−	Borderline

Abbreviations: MNBI/AET, ratio of mean nocturnal baseline impedance to acid exposure time; MNBI, mean nocturnal baseline impedance; AET, acid exposure time; RE, reflux episode; SA, symptom association; IEM, ineffective esophageal motility; HH, hiatal hernia. Positive (+) symptom association (SA) was defined as a symptom index > 50% and/or a symptom association probability > 0.95. pH-MII parameters of 21 patients who were initially classified as borderline and/or supportive according to the Lyon Consensus 2.0 criteria and subsequently reclassified as having definitive GERD based on an MNBI/AET ratio cut-off value of ≤624.28. In the supportive group, patients with IEM or a HH < 3 cm on manometry are indicated in parentheses.

## Data Availability

The data presented in this study are available on request from the corresponding author.

## Abbreviations

The following abbreviations are used in this manuscript:

AET	Acid Exposure Time
AUC	Area Under The Curve
CI	Confidence Interval
DCI	Distal Contractile İntegral
FH	Functional Heartburn
EoE	Eosinophilic esophagitis
HREM	High-Resolution Esophageal Manometry
GERD	Gastroesophageal Reflux Disease
GI	Gastrointestinal
HH	Hiatal Hernia
LA	Los Angeles
LES	Lower Esophageal Sphincter
mmHg	Millimeters of mercury
MNBI	Mean Nocturnal Baseline Impedance
MII-pH	Multichannel Intraluminal Impedance–pH
NCCP	Non-Cardiac Chest Pain
NERD	Non-Erosive Reflux Disease
IEM	Ineffective Esophageal Motility
IQR	Interquartile Range
IRP	Integrated Relaxation Pressure
PSPW	Post-Reflux Swallow-Induced Peristaltic Wave
PPIs	Proton Pump Inhibitors
RE	Reflux Episode Count
ROC	Receiver Operating Characteristic
SAP	Symptom Association Probability
SI	Symptom Index
Ω	Ohm
